# Supplementary material for: Among the world’s smallest vertebrates: a new miniaturized flea-toad (Brachycephalidae) from the Atlantic rainforest
Source: PeerJ. 2024 Oct 25;12:e18265. doi: 10.7717/peerj.18265 (PMC11514764; doi:10.7717/peerj.18265)
Supplement: Supplemental Information 5 [file peerj-12-18265-s005.docx]

**Supplementary material**

**AMONG THE WORLD'S SMALLEST VERTEBRATES: A NEW MINIATURIZED FLEA-TOAD FROM THE ATLANTIC RAINFOREST**

Luís Felipe Toledo, Lucas Machado Botelho, Andres Santiago Carrasco-Medina, Jaimi A. Gray, Julia R. Ernetti, Joana de Moura Gama, Mariana L. Lyra, David C. Blackburn, Ivan Sergio Nunes, Edelcio Muscat

**Table S1.** Uncorrected p-distances for the Palumbi 16S fragment between (A) individuals of *Brachycephalus dacnis* and the sister species *B. hermogenesi*; and (B) between species of *Brachycephalus* spp. (mean distances). Distances within species (A) or within species groups (B) are highlighted in gray. Distances between *B. dacnis* and other species are in bold.

**Table provided as .xlsx file**

**Table S2.** CT-scanning parameters used for each *Brachycephalus* spp. individual.

**Table provided as .xlsx file**

**Table S4.** Samples included in the phylogenetic inference.

**Table provided as .xlsx file**

**Table S3.** Amplified fragments, primers, and PCR protocols used in this study. Total reaction volume of 25 μL, containing 1X AmpliTaq Gold® 360 Master Mix (Life Technologies), 10 μM of each primer and < 1μg/reaction of template DNA extracted.

| **Fragment** | **Primers** | **PCR program** |
| --- | --- | --- |
| **upstream** | **12s-L13** -forward  (TTA GAA GAG GCA AGT CGT  AAC ATG GTA)  Feller and Hedges (1998) | 95°C (3 min), followed by 36 cycles of denaturation at 95°C (20 s), annealing at 50°C (20 s), extension at 68°C (1 min and 20s), and a final extension at 68°C (3 min) |
|  | **16S-H10** -reverse  (TGC TTA CGC TAC  CTT TGC ACG GT)  Hedges (1994) |  |
| **dowstream** | **16s-AR** -forward  (CGC CTG TTT ATC AAA AAC AT)  Palumbi et al. (1991) | 95°C (10 min), followed by 36 cycles of denaturation at 95°C (30 s), annealing at 55°C (30 s), extension at 72°C (1 min), and a final step at 72°C (7 min) |
|  | **16s-BR** -reverse  (CCG GTC TGA ACT CAG ATC  ACG T)  Palumbi et al. (1991) |  |

**References**

Feller, A., and S.B. Hedges. 1998. Molecular evidence for the early history of living amphibians. Molecular Phylogenetics and Evolution 9: 509–516.

Hedges, S. B. 1994. Molecular evidence for the origin of birds. Proceedings of the National Academy of Sciences of the United States of America 91:2621–2624.

Palumbi, S.R., A. Martin, W.O. McMillan, L. Stice, and G. Grabowski. 1991. The simple fool’s guide to PCR, version 2.0. Privately published.

**Figure S1.** Bayesian phylogenetic inference of *Brachycephalus* ssp. based on the mitochondrially encoded 16S rDNA fragment. Black dots indicate fully supported clades (Posterior probability, PP => 99). White dots indicate PP >0.95. Node supports below pp=0.95 are not shown.

**
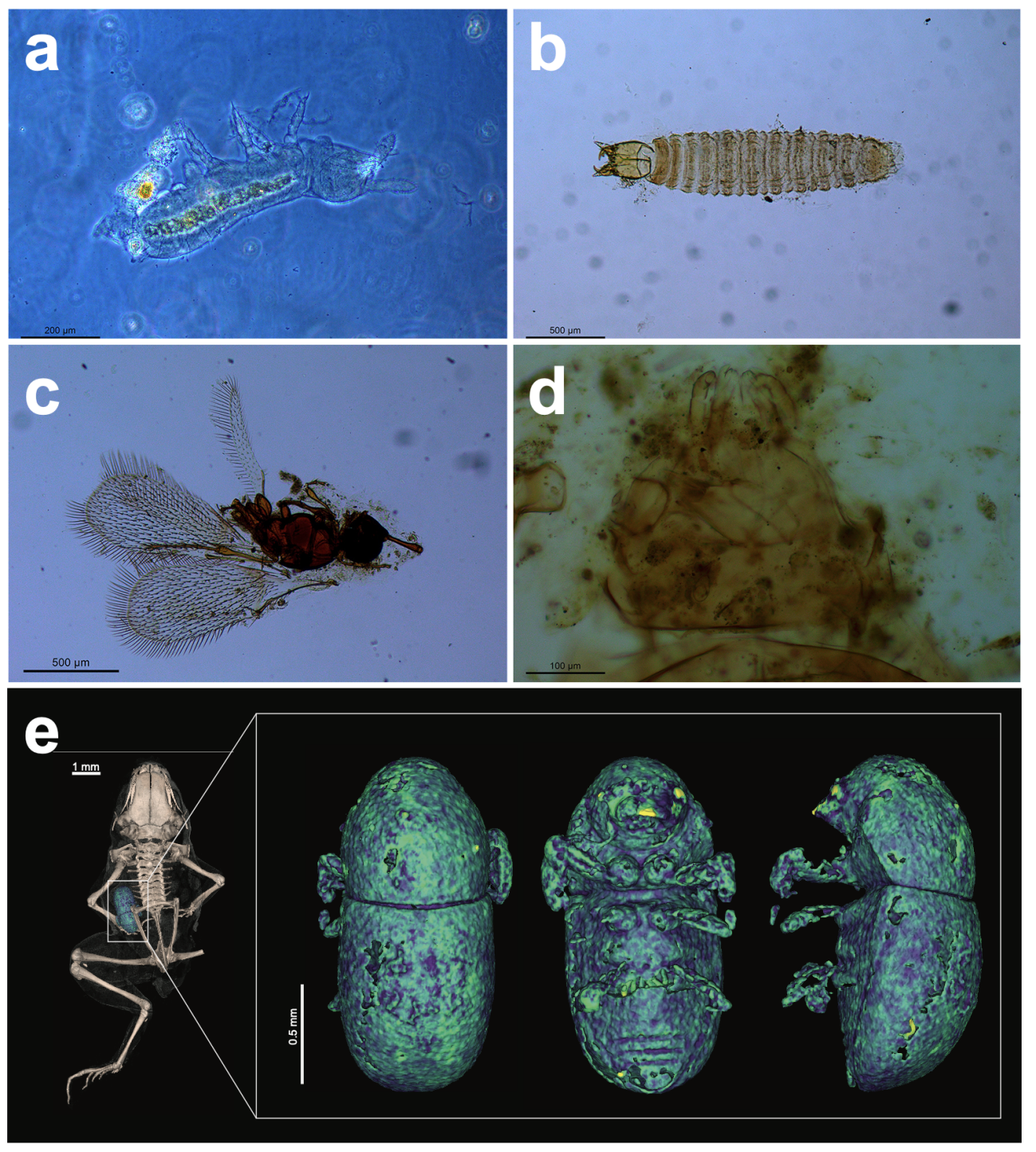
Figure S2.** Prey items of *Brachycephalus dacnis*: (a) Hexapoda, Collembola; (b) Hexapoda, Coleoptera; (c) Hexapoda, Hymenoptera, Chalcidoidea; (d) Acari, Sarcoptiformes; and (e) an Anobiinae beetle (Coleoptera: Ptinidae) identified after the CT-scan imaging of one specimen (photo credit: a-d: Ivan Nunes, and e: David C. Blackburn).

**Appendix I.** Examined specimens (besides those presented in Appendix II) and museum acronyms.

- CCLZU: Coleção Científica do Laboratório de Zoologia da Universidade de Taubaté, SP
- HCLP-A: Coleção de Anfíbios do Laboratório de Herpetologia, Instituto de Biociências, Universidade Estadual Paulista, Campus do Litoral Paulista, SP
- MNRJ: Museu Nacional, Universidade Federal do Rio do Janeiro, RJ
- MZUFV: Museu de Zoologia Joao Moojen de Oliveira, Universidade Federal de Viçosa, MG
- MZUSP: Museu de Zoologia, Universidade de São Paulo, SP
- ZUEC-AMP: Museu de Diversidade Biológica (MDBio), Universidade Estadual de Campinas, SP
- ZUFMS: Coleção Zoológica do Instituto de Biociências da Universidade Federal do Mato Grosso do Sul, MS

*Brachycephalus didactylus* species group:

*Brachycephalus didactylus*: Rio de Janeiro, Paulo de Frontim (MNRJ 4067–73 paratypes; ZUEC-AMP 1132–35, 10825 topotypes, MZUSP 64812, 94620). Rio de Janeiro, Cachoeiras de Macacu, Parque Estadual dos Três Picos (CFBH 40835, 40837, 40844)

*Brachycephalus dacnis*: São Paulo, Ubatuba (ZUEC-AMP 24982 holotype, 24978–79; 24981; 24984; 25270; 25272–75; 25612 paratypes, HCLP-A 267–68 paratypes).

*Brachycephalus hermogenesi*: São Paulo, Ubatuba (ZUEC-AMP 9715 holotype, 9716–19, 9721–24 paratypes), São Paulo, Salesópolis, Estação Biológica de Boraceia (CFBH 20125, 20128, MZUSP 138347).

*Brachycephalus izecksohni*: Paraná, Quaratuba (MNRJ 76259–60 paratypes).

*Brachycephalus pulex*: Bahia, Camacan, Serra Bonita (CFBH 39379).

*Brachycephalus ephippium* species group:

*Brachycephalus darkside*: Minas Gerais, Ervália (MZUFV 16636, holotype, MZUFV 15557–15561, 15565–15571, 15716–15721, 16491, 16579, 16627–16628,16631–16633, 16634–16635, 16780 paratypes, UFMG 19522 paratype, MNRJ 91327 paratype); Miradouro (MZUFV 2897, 6658–6660 paratypes).

*Brachycephalus ephippium*: Rio de Janeiro, Itatiaia (MZUFV 4161, MNRJ 52427–40); Nova Friburgo (MNRJ 39342–43); Rio de Janeiro (MNRJ 30919–29, 40782–807); Teresópolis (MNRJ 51580–83).

*Brachycephalus garbeanus*: Rio de Janeiro, Nova Friburgo (MZUSP 0811 lectotype, MNRJ 17440–41, 25390–400, 67498).

*Brachycephalus ibitinga*: São Paulo, Santo André (ZUEC-AMP 444).

*Brachycephalus margaritatus*: Rio de Janeiro, Petrópolis (MNRJ 85300–396).

*Brachycephalus rotenbergae*: São Paulo, São José dos Campos (HCLP-A 260 holotype, HCLP-A 78-83, 284-287 paratypes, CCLZU 1274, 1278-1279 paratypes, ZUFMS-AMP 13647–13649 paratypes, HCLP-A 281-283).

*Brachycephalus vertebralis* species group:

*Brachycephalus alipioi*: Espírito Santo, Castelo (MZUFV 5877–5882).

*Brachycephalus bufonoides*: Rio de Janeiro, Nova Friburgo (MZUSP 1459 lectotype).

*Brachycephalus crispus*: São Paulo, Cunha (ZUEC-AMP 24448, 24450 paratypes).

*Brachycephalus nodoterga*: São Paulo, São Paulo (MZUSP 0975 holotype).

*Brachycephalus pitanga*: São Paulo, São Luís do Paraitinga (MNRJ 60790–93 paratypes).

*Brachycephalus toby*: Brazil, São Paulo, Ubatuba (MNRJ 76382–83 paratypes, ZUEC-AMP 24443, 24449 paratypes).

*Brachycephalus vertebralis*: Rio de Janeiro, Paraty (MNRJ 11098 holotype, 89199–201).

*Brachycephalus pernix* species group:

*Brachycephalus brunneus*: Paraná, Campina Grande do Sul (MNRJ 40289–91, paratypes, CFBH 7880 paratype).

*Brachycephalus leopardus*: Paraná, Tijucas do Sul (ZUEC-AMP 25322).

*Brachycephalus pernix*: Paraná, Quatro Barras (MNRJ 17343 holotype, MNRJ 17328–42, 17427–28, paratypes).

*Brachycephalus tridactylus*: Paraná, Guaraqueçaba (MNRJ 87908–910).

**Appendix II.** Additional material examined. For CT-scan data, we cite the MorphoSource ARK (complete vouchering numbers will be provided upon the acceptance of the manuscript).

*Brachycephalus albolineatus*: MHNCI 10274 (ark:/87602/m4/M14739).

*Brachycephalus brunneus*: CFBH 7880 (paratype).

*Brachycephalus clarissae*: MNRJ 89562 (ARK).

*Brachycephalus coloratus*: MHNCI 10274 (ARK).

*Brachycephalus curupira*: MHNCI 10285 (ARK).

*Brachycephalus didactylus*: ZUEC-AMP 1132–3, 10825 (topotypes); MZUSP 64812, 94620 (topotypes); CFBH 40835, 40837, 40844; MCZ A-109799 (ARK).

*Brachycephalus ephippium*: MCZ A-108660 (ARK), 108670 (ark:/87602/m4/M16126); UF-Herp-72725 (ARK).

*Brachycephalus hermogenesi*: ZUEC-AMP 9715 (holotype), 9716–9, 9721–4 (paratypes), 23204, 24980 (ARK); MZUSP 138347; CFBH 20125, 20128.

*Brachycephalus margaritatus*: MNRJ 80998 (ARK).

*Brachycephalus nodoterga*: MCZ A-108602 (ark:/87602/m4/M16094).

*Brachycephalus pernix*: MHNC 19809 (ARK).

*Brachycephalus pitanga*: MNRJ 60793 (ARK).

*Brachycephalus pulex*: CFBH 39379 (topotype).

*Brachycephalus sulfuratus*: ZUEC-AMP 16620, 19931; DZUP 151 (ARK), 152 (ARK).

*Brachycephalus vertebralis*: MNRJ 89201 (ARK).

*Ischnocnema nasuta*: USNM 23574 (ark:/87602/m4/381433)
